# Nightmare case of repeated hemorrhagic pancreatitis due to pancreatic head cancer in the acute stage after two-stage lung transplantation

**DOI:** 10.1016/j.jhlto.2025.100245

**Published:** 2025-03-11

**Authors:** Tatsuya Hayashi, Shin Tanaka, Tsuyoshi Ryuko, Yasuaki Tomioka, Kentaroh Miyoshi, Mikio Okazaki, Seiichiro Sugimoto, Shinichi Toyooka

**Affiliations:** a Department of General Thoracic Surgery, Okayama University Hospital, Japan; b Department of General Thoracic Surgery and Organ Transplant Center, Okayama University Hospital, Japan

**Keywords:** Two-stage lung transplantation, Sequential single-lung transplantation, Immunosuppressive therapy, Perioperative pancreatitis, Malignancy screening

## Abstract

In Japan, due to the shortage of deceased organ donors, the use of two-stage lung transplantation (LTx), specifically sequential single-lung transplants (SSLTs), has been increasing. This approach starts immunosuppressive therapy after the first transplantation, which increases the risk of malignancies. Here, we present a case of fatal perioperative hemorrhagic pancreatitis caused by pancreatic head cancer following SSLTs. This case highlights significant challenges in diagnosis and treatment.

## Case presentation

**Figure 1:**
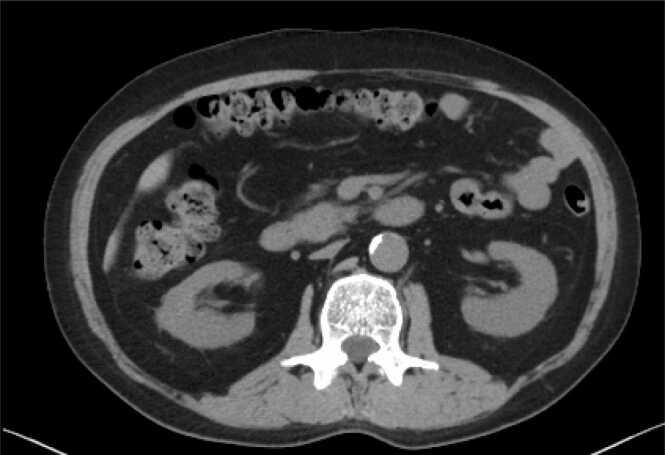
Preoperative Computed tomographic (CT) scan before the second lung transplantation. No obvious tumor lesions were detected in the pancreatic head.

A 57-year-old man underwent a right single-lung transplantation from a brain-dead donor three years earlier for idiopathic interstitial pneumonia. He had a five-year history of steroid and cyclosporine use. Postoperatively, the patient was treated with standard immunosuppressive therapy, including tacrolimus, mycophenolate mofetil (MMF), and steroids, per normal institutional protocols. Two years after the initial transplant, he was listed for re-transplantation due to chronic lung allograft dysfunction and underwent a left single-lung transplantation one year later. Preoperative evaluations: including vital signs, tumor markers (CEA=4.84), chest-abdominal computed tomography (CT) scans (Figure 1), and intraoperative findings were unremarkable. Postoperatively, standard immunosuppressive therapy was administered alongside basiliximab on the first and third postoperative days.

**Figure 2:**
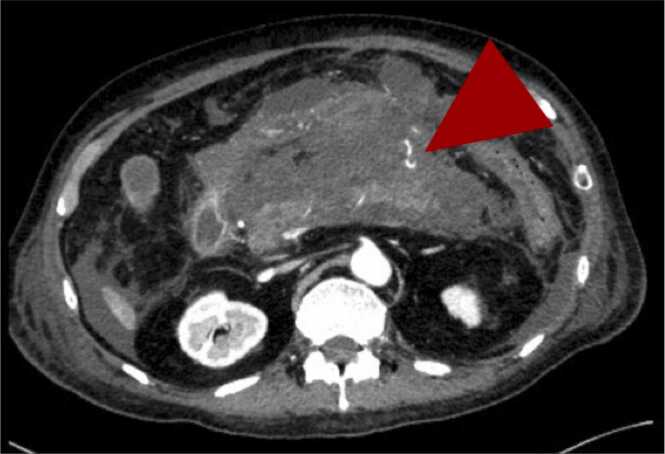
Contrast-enhanced abdominal Computed tomographic (CT) images reveal an extensive area of inflammation surrounding the pancreas. Extravasation of contrast material, indicated by arrows, suggests active bleeding.

**Figure 3:**
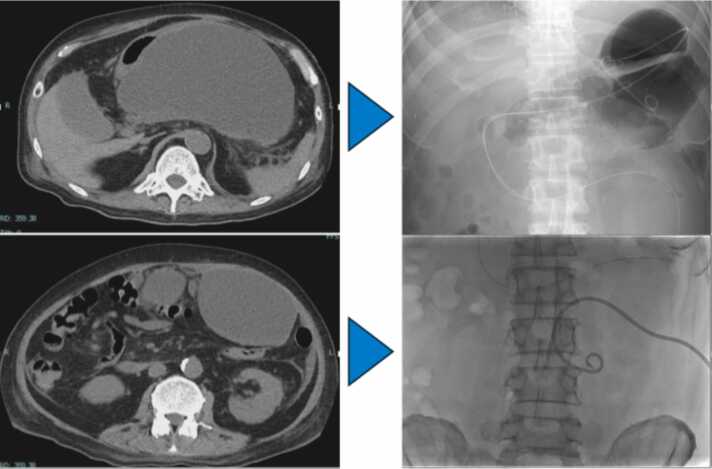
Abdominal Computed tomography (CT) and fluoroscopic images demonstrate multiple large cystic lesions in the abdominal cavity, including dorsal to the stomach and near the anterior abdominal wall. These lesions were treated using transgastric and percutaneous drainage.

After initial postoperative progress, the patient’s condition suddenly deteriorated by postoperative day 10, with the onset of acute abdominal pain, elevated pancreatic enzymes, and inflammation. Abdominal CT indicated pancreatitis. Fasting and nafamostat mesylate were initiated, but the episodes recurred. Two weeks after onset, worsening abdominal pain and contrast-enhanced CT findings of extravasation prompted interventional radiology (IVR) treatment (Figure 2). Neither the source of bleeding nor an obstruction could be identified, even after Endoscopic Ultrasonography (EUS). Suspecting drug-induced pancreatitis, tacrolimus was switched to cyclosporine, and antibiotics were adjusted. Despite persistent recurrences and the formation of multiple intra-abdominal cysts requiring drainage, the etiology of hemorrhagic pancreatitis remained undetermined (Figure 3).

During the fifth relapse, gastrointestinal endoscopy and EUS revealed a pancreatic head mass. A biopsy confirmed pancreatic cancer with obstructive pancreatitis. Symptom relief was achieved through stent placement in the pancreatic duct, and the patient was discharged three months postoperatively. Chemoradiotherapy was discontinued due to adverse reactions. One year postoperatively, the patient succumbed to pancreatic head cancer. No graft dysfunction occurred throughout this period.

## Discussion

Single-lung and bilateral lung transplantation are the primary surgical options for lung transplants. While bilateral transplants offer better long-term outcomes, single-lung transplants are favored in Japan due to donor shortages and reduced surgical invasiveness.^1^ Recently, two-stage sequential single-lung transplantation (SSLTs) is a viable strategy for high-risk patients, such as the elderly or those with comorbidities.^2^ SSLTs include planned and unplanned cases, like this one with contralateral surgery for chronic rejection. While outcomes match bilateral transplantations, challenges include anatomical complexities during the second surgery and increased malignancy risks from intensified immunosuppressive therapy during two perioperative periods.^3^

Generally, Malignancy risks are particularly high in lung transplant recipients, with a standardized incidence rate of 3–5 times that of the general population within three years post-transplant. Contributing factors include the lungs' exposure to environmental pathogens and the need for aggressive immunosuppressive therapy to manage rejection.^4^ In patients with SSLTs, the need for two rounds of high-dose immunosuppressive therapy in the acute postoperative phase may further increase the risk of malignancy. Currently, reports on SSLTs are limited due to the small number of cases.^5^ Among them, however, neither provides results or discussions on long-term prognosis or causes of death, including malignancies. Nevertheless, concerns regarding malignancies and immunological complications associated with immunosuppressive therapy have been raised, highlighting the need for further research. This case is infrequent, as the malignancy, which was undetectable preoperatively, rapidly progressed during the acute postoperative phase to the point of causing obstructive hemorrhagic pancreatitis. To our knowledge, there have been no previous reports of such rapid tumor growth with symptomatic presentation in the acute postoperative period. This case highlights the importance of perioperative screening and management strategies in patients undergoing sequential single-lung transplantation.

Enhanced malignancy screening is crucial to mitigate these risks.^6^ Several guidelines recommending malignancy screening, including those from the American Society of Transplant, have been reported. However, these recommendations do not include the measurement of tumor markers other than those for liver and prostate cancer, nor do they advocate for high-resolution imaging studies such as CT scans.^7^ For SSLTs patients, incorporating annual malignancy screening, such as the measurement of tumor markers and CT scans, into standard post-transplant care could enable early detection and intervention, improving outcomes for this high-risk population. In patients undergoing SSLTs, including this case, postoperative management should have been conducted with malignancy in mind, even during the acute postoperative phase.

## Patient consent

The authors confirm that appropriate patient consent to publish this case report was received.

## Disclosure statement

The authors declare that they have no known competing financial interests or personal relationships that could have appeared to influence the work reported in this paper. This work was supported by a Grant-in-Aid for Young Scientists(Start-up) (Grant No. 24K2348906) from the Japan Society for the Promotion of Science.
